# Human Rabies by Secondary Transmission in Argentina, 2021

**DOI:** 10.3390/diseases10010017

**Published:** 2022-03-18

**Authors:** Soledad Firpo, María Guadalupe Piccirilli, Rogelio Urizar, Nicolas Vitta, Stella Maris Hirmas Riade, Constanza Leguizamón, María Lorena Vico, Gustavo Martínez, Fernando J. Beltrán, Daniel M. Cisterna

**Affiliations:** 1 Hospital Municipal “Raúl Caccavo”, Coronel Suárez B7540, Provincia de Buenos Aires, Argentina; soledadfirpo@hotmail.com (S.F.); rourizar@yahoo.com.ar (R.U.); nicovittal@gmail.com (N.V.); 2 Servicio de Neurovirosis, Departamento de Virología, Instituto Nacional de Enfermedades Infecciosas, ANLIS “Carlos G. Malbran”, Ciudad Autónoma de Buenos Aires 1282AFF, Argentina; piccirilli.guadalupe@gmail.com (M.G.P.); stellamh@gmail.com (S.M.H.R.); 3 Instituto de Zoonosis “Luis Pasteur”, Ciudad Autónoma de Buenos Aires C1405DCD, Argentina; connieleguizamon@gmail.com (C.L.); ferbelt@hotmail.com (F.J.B.); 4 Departamento de Zoonosis Urbanas, Avellaneda B1870, Provincia de Buenos Aires, Argentina; lorenavico@yahoo.com.ar (M.L.V.); zoonosisurbanas@gmail.com (G.M.)

**Keywords:** human rabies, bats, feline rabies

## Abstract

Rabies is a zoonotic disease caused by the rabies virus (RABV) that causes fatal encephalitis in mammals. Bats can transmit the disease to urban canines and felines, which rarely infect humans, establishing a secondary link. The last case of human rabies in Argentina was transmitted by a dog in 2008. We present the first case of human rabies originating from an insectivorous bat, *Tadarida brasiliensis*, transmitted by a feral cat in Buenos Aires province, Argentina.

## 1. Introduction

The rabies virus is an RNA virus (RABV) that belongs to the *Rhabdoviridae* family, genus *Lyssavirus*. It is the only one that has been detected in the American continent [1]. RABV circulates in several species of bats, in terrestrial mammals such as canids, felids, and mustelids, among others, and in primates of the species *Callithrix jacchus* [2]. Occasionally, aerial species can transmit the disease to terrestrial ones, an event that is known as a primary transmission or spillover, documented more frequently in the urban or peri-urban environment in cats and dogs, although it can also be detected in wild areas [3]. These animals are very rarely able to transmit the disease to humans, establishing a secondary link, which, if not treated promptly, will culminate in fatal encephalitis [4].

In countries where domestic rabies could be controlled by vaccination programs, bats have become an increasingly frequent source of infection. In Argentina, between 2013 and 2020, a total of 821 cases of animal rabies were notified to the National Epidemiological Surveillance System [5]: 82% of the cases corresponded to insectivorous bats, 12% were related to the hematophagous bat *Desmodus rotundus*, 5% to dogs and other terrestrial wild animals, and 1% to cats.

Human rabies transmitted by bats is very rare, so that healthcare workers and the general population may not be conscious of the risks of direct injury. Additionally, many people are unaware that they can acquire rabies through their pets that may be carrying the disease due to having had previous contact with one of these reservoir species. In this study, a case of human rabies of bat origin transmitted by a feral cat is described. The epidemiological and sanitary situation of rabies in the area of the case is discussed.

## 2. Case Report

**Figure 1:**
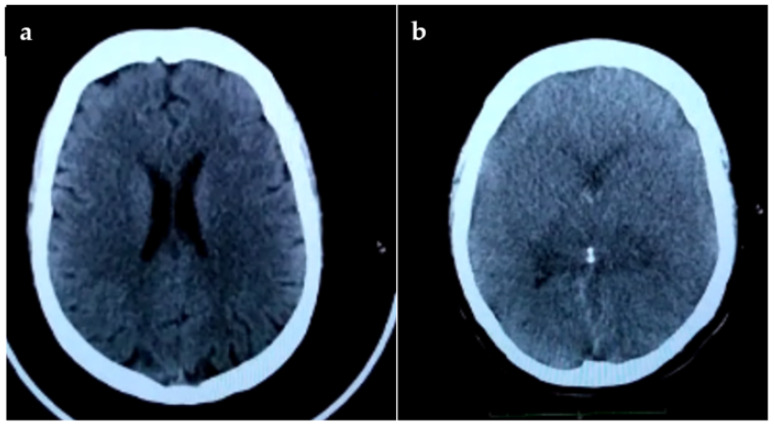
(**a**) Computed tomography does not show significant changes in the cerebral ventricles; (**b**) This image demonstrates frank cerebral edema with collapse of the ventricles and effacement of the fissures.

A 33 year old patient was admitted to the “Dr. Raúl Caccavo” Municipal Hospital, Coronel Suárez, Buenos Aires province, on 22 April 2021 with paresthesias and loss of strength in both hands of four days of evolution. The previous day, she began with difficulty swallowing, and with a feeling of shortness of breath. At the time of the consultation, she presented muscle spasms in the upper limbs, chest, and face with constant movements and reactions to sound and light stimuli. The patient showed gait stability, without alterations in the lower limbs, no focal deficits, no meningeal signs. She had involuntary movements of the neck of the contorting type. A simple computed tomography of the brain (CT) was performed, which did not show alterations (Figure 1a). An examination of the cerebrospinal fluid (CSF) showed a clear, colorless fluid, normal glucose, elevated protein, and a normal white cell count.

The next day, the patient presented gait disturbances, instability, urinary sphincter incontinence, and began with hypotension, intense sweating, fever and vomiting, and an episode of lividity. The patient was anuric with a need for dialysis and for the involuntary movements to be moderated. It was decided to transfer her to the Intensive Care Unit (ICU) and to perform sedation and motor respiratory assistance. Later, she began with a constant fever of 39–40 °C, and antibiotic treatment with Piperacillin-Tazobactam was indicated. Molecular tests in the CSF for herpes viruses 1, 2 and 6, cytomegalovirus, Epstein–Barr virus, and enterovirus were negative. Additionally, the culture for common bacterial pathogens in the CSF was also negative.

Twelve days after admission (4 May 2021), a second brain CT scan was performed and severe cerebral edema was observed (Figure 1b).

**Figure 2:**
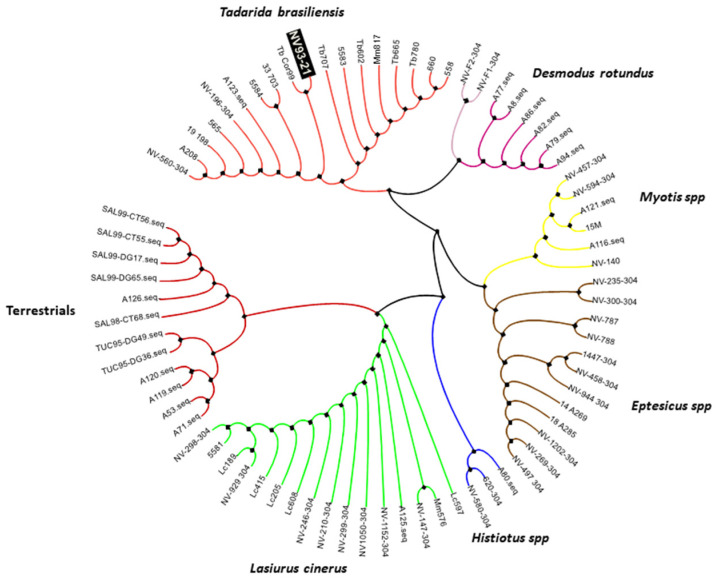
Genetic typing of partial nucleoprotein gene (264nt) from Argentinean rabies virus strain. The phylogenetic tree was constructed using the maximum-likelihood method, and bootstrap values were calculated from 1000 replicates with the software MEGA X (https://www.megasoftware.net/; Accessed: 15 May 2021). The tree obtained was edited using the online tool iTOL v6 (https://itol.embl.de/). Representative RABV sequences were included to define the groups of circulating reservoir species in Argentina [8]. The sample NV93-21 (underlined in the image) corresponds to the case under study (GenBank accession number OM909029).

That same day, her husband remembers that the patient had been bitten by a street cat, which the patient wanted to feed, 42 days before the onset of symptoms (3 June 2021). The bite was on the right hand, on the index, and middle fingers. The patient did not consult the doctor for this bite because she only had signs of local phlogosis, little pain, and immediate healing. She did not receive post-exposure treatment for rabies, and the animal was never located. Given the suspicion of human rabies, on 05/10/21 samples of nuchal skin biopsy, CSF, saliva, and a conjunctival swab were taken to perform an LN34 pan-lyssavirus real-time assay (LN34 RT-qPCR) designed by the Centers for Disease Control and Prevention, Atlanta, USA [6] along with a serum to search for antibodies by ELISA test (Platelia Rabies II Kit, Bio Rad, Marnes La Coquette, France), all being negative for RABV. Nineteen days after her hospitalization, the patient died (13 May 2021). Necropsy samples were taken from the brain, Ammon’s horn, and cerebellum. The RT-qPCR and direct fluorescent antibody test (dFA) [7] confirm the presence of rabies virus. Partial genetic sequencing of the rabies virus nucleoprotein gene using primers 304 and 10G identified the variant of the insectivorous bat *Tadarida brasiliensis* (Figure 2) [8]. The local and regional Zoonosis Department carried out a ring rabies vaccination on 1417 felines and canines older than three months within a radius of 500 m from the patient’s home. The timeline of the case is depicted in Supplementary Figure S1.

**Figure 3:**
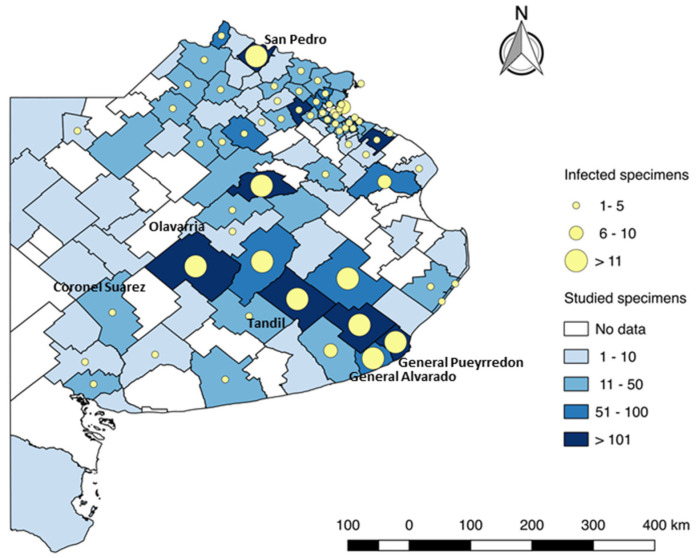
Distribution of rabies in insectivorous bats in the province of Buenos Aires, 2018–2021. The map was constructed using QGis 3.22 (https://www.qgis.org/es/site/; Accessed: 15 September 2021) with the information provided by the Departamento de Zoonosis Urbanas, Avellaneda, Provincia de Buenos Aires through animal rabies surveillance.

There are previous reports of rabies detection in insectivorous bats in the province of Buenos Aires (Figure 3). Between 2014 and September 2020, rabies was detected in approximately 6% of the total chiropterans studied. In the municipality of Coronel Suárez, cases of rabies have been detected in *Tadarida brasiliensis* and *Myotis* sp. bats without interruption since 2018 [9]. According to available provincial information, the annual rate of veterinary rabies vaccination coverage achieved with public resources remained on average at 14.5–15.0% until 2019. In August 2020, there was a marked decrease (2.9%) attributable to movement restrictions and strict social isolation associated with the COVID-19 pandemic [9].

## 3. Discussion

Cases of human rabies with secondary transmission are not frequently reported. In Latin America, Kotait, et al. [10] have described only eight cases between 2001 and 2012. In each case, the vector identified was a cat, and a rabies variant associated with a local bat species was recognized. More recently, in Brazil, Colombia and Perú, the importance of the cat as an intermediate species in the transmission of rabies maintained by hematophagous bats has been highlighted [11].

Since the control of canine rabies in the USA, cats have become the most common domestic animal to contract rabies and even exceed the number of rabid dogs diagnosed. The role of free-roaming or feral cats in the transmission of rabies is unknown. Cases of feline rabies are usually associated with variants maintained by terrestrial wild species such as raccoons or skunks [12]. In contrast, in Brazil between 2002 and 2016, 24 positive cases were reported in the Sao Pablo State (eighteen dogs and 16 in cats), and genetic typing indicated that the disease had been transmitted by bats [13]. Similarly, in Argentina, the totality of feline rabies cases diagnosed between 2013 and 2020 originated in insectivorous bats. These data suggest that cats are likely to play an important role in the secondary transmission of bat-associated rabies variants to humans in Latin American countries. The predatory role of the domestic cat in urban and suburban areas in relation to small birds and mammals is known. Observational records of cat attacks and, more recently, traces of their DNA in uropatagium membrane lesions have been observed in bat rescue centers [14].

Carrying out a vaccination program in feral cats and dogs is difficult to implement, requiring the use of traps as well as physical and human resources. It is important to encourage responsible pet ownership by reducing the number of unwanted and abandoned cats, increasing the programs available for ethical adoption, and improving vaccination rates for all domestic animals. Another aspect to consider and publicize is the danger which the people who feed them are exposed to, people who, in the event of a bite episode, underestimate the need to consult medical services.

Fortunately, in Argentina, cases of human rabies are very rare today. However, without any close history of exposure and nonspecific symptoms, it is a real challenge to reach an early diagnosis. During the study of this patient, a wide variety of presumptive diagnoses were proposed, which involved carrying out a large number of studies and laboratory tests that did not have positive results: botulism, tetanus, Miller–Fisher syndrome, intoxication by drugs or toxic substances, metabolic diseases, autoimmune disease, and atypical presentation of COVID-19. Finally, the impact of the pandemic, which has produced an evident decrease in public and private vaccination and animal control mechanisms, should be highlighted.

## 4. Conclusions

A case of human rabies represents a weakness of the health system since the disease can be prevented. This must be addressed in an interdisciplinary and intersectoral manner with the “One Health” approach, which involves the human, animal and environmental disciplines and the public and private systems.

## Data Availability

The data that support the findings of this study are available on request to the corresponding author.

## Supplementary Materials

The following supporting information can be downloaded at: https://www.mdpi.com/article/10.3390/diseases10010017/s1, Figure S1: Timeline of a case of human rabies that occurred in the province of Buenos Aires, Argentina, 2021.
