# Supplementary material for: Human Rabies by Secondary Transmission in Argentina, 2021
Source: Diseases. 2022 Mar 18;10(1):17. doi: 10.3390/diseases10010017 (PMC8947700; doi:10.3390/diseases10010017)
Supplement: Supplementary file 1 [file diseases-10-00017-s001.zip › diseases-1619594-supplementary.pdf]

Supplementary Figure S1. Timeline of a case of human rabies that occurred in the province of Buenos Aires, Argentina, 2021

03/06/21 — **The patient had been bitten by a street cat.**

04/22/21 — 33-year-old patient who was admitted to the Dr. Raúl Caccavo Municipal Hospital with paresthesias and loss of strength in both hands.

04/23/21 — It was decided to transfer her to the Intensive Care Unit (ICU) and to perform sedation and motor respiratory assistance

05/04/21 — Computed tomography scan was performed and severe cerebral edema was observed

05/10/21 — A relative of the patient remembers that he was bitten by a stray cat. **Ante Mortem rabies diagnosis was started.**

05/13/21 — Nineteen days after her hospitalization, the patient died. **Post Mortem rabies diagnosis was started.**

Ante Mortem Rabies Diagnosis

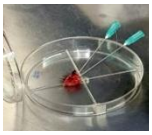

Nuchal skin biopsia

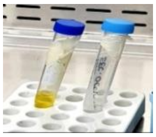

Serum, Cerebro spinal fluid

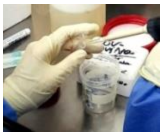

Salive

Post Mortem Rabies Diagnosis

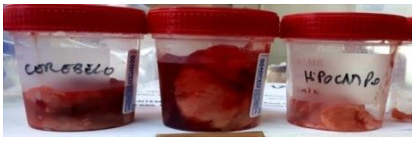

Necropsy samples: brain, Ammon's horn, and cerebellum

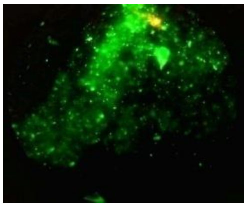

Rabies detection by direct Fluorescent antibody test

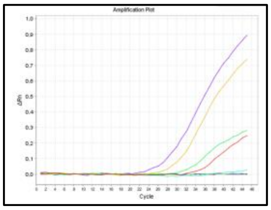

Rabies detection by LN34 pan-lyssavirus real-time assay
